# Dr. Clough's Case of Exomphalocele

**Published:** 1811-07

**Authors:** H. G. Clough

**Affiliations:** Berner's Street


*To the Editors of the Medical and Physical Journal.*

Gentlemen,

An extraordinary case of Exomphalocele having occurred (about twelve months since) to a new born male infant, I embrace this opportunity of communicating the same to you, that you may give it a place in your periodical work, should it meet your approbation.

April 15, 1810.—John Brown, residing at 2, Upper Charlton Street, Fitzroy Square, was admitted as a patient on the medical list of the Endeavour Society. I first visited this child about a fortnight after its birth, and found a rupture, measuring in diameter four inches, and in height at the centre one inch and a half, covered by the omentum and peritoneum; on which surface there was an uniform unctuous exudation; this rupture was surrounded by the umbilicus licus bearing the funis on the left, the edges were in a state of inflammation. I requested Mr. Clough, of Norton Street, Surgeon of the Establishment, might be sent for, and we agreed in opinion that it would be more secure to bring the parts together by the first intention; which plan we adopted, covering the softer parts with dossils of lint. About a week after very high inflammation arose, and we were under the necessity of leaving off the adhesive pressure, and had recourse to a weak solution of the superacetate of lead, with a moderate bandage round the body. This plan was pursued until the 26th of May, when the part was reduced to about the size of a sixpence, and the child was sent into the country, with every prospect of doing well.

I am, Gentlemen,
Your humble Servant,
H. G. CLOUGH, M. D.

*Berner's Street, 11th May,* 1811.

